# A 3‐year controlled clinical trial comparing high‐translucency zirconia (cubic zirconia) with lithium disilicate glass ceramic (e.max)

**DOI:** 10.1002/cre2.790

**Published:** 2023-10-03

**Authors:** Hiba A. Fawakhiri, Souad Abboud, Shaza Kanout

**Affiliations:** ^1^ Department of Operative Dentistry Damascus University Damascus Syria; ^2^ Department of fixed Prothdontiscs Damascus University Damascus Syria

**Keywords:** e‐max, gingivitis, translucent zirconia, veneers

## Abstract

**Introduction:**

The rate of clinical success in veneers, under esthetics, has achieved a range of 18 months to 20 years. In a plethora of studies, it registers a success rate reaching 75% and even 100%. The most common type of glass ceramics used in ceramics is the vitreous lithium disilicate crystal‐reinforced material, e.max®. Recent studies focus on “polycrystalline ceramic use” in manufacturing veneers, as it possesses a stronger structure and different enabling manufacturing schemes.

**Objectives:**

This research aims at comparing e.max and the high‐translucent Cubic Zirconia. Such comparison is administered to veneers manufacturing: esthetic (surface and edge, and staining and color matching), functional (crack and fracture, contact point, and patient satisfaction), and biological (posttreatment vitality and hypersensitivity, and periodontal response).

**Materials and Methods:**

The research sample consisted of 60 veneers, divided into two groups: cubic zirconia and e.max. The sample included 2 males (16.6%) and 10 females (83.3%), with age ranging from 25 to 37 years. Patients were thoroughly diagnosed and treated and included in this study based on certain inclusion–exclusion criteria. Hickel's 2010 criteria were utilized to examine and observe the clinical aspect of veneers during intervals of 1 week, 3 months, 1 year, and 3 years.

**Results:**

No significant differences were traced across the groups for all the variables, with a *p*‐value being greater than 0.05. The *e.max* group revealed better clinical results compared to the *cubic zirconia* one regarding esthetics and tooth translucency. Nonetheless, the results showed a merely slight increase in hypersensitivity in the e.max group.

**Conclusion:**

Within the limitation of an extensive follow‐up period, we can conclude that there is no difference between cubic zirconia and e.max (*p* > 0.05), where e.max and *cubic zirconia* veneers have the same characteristics in terms of the following. The characteristics of the aspects examined were esthetic, functional, and biological.

## 1 INTRODUCTION

Preservation of tooth structure and prevention of needless tooth structure removal are basic and important principles in prosthodontics. Striving to achieve the cosmetic aspect is also a basic requirement, especially in the modern world. Veneers can satisfy both of the aforementioned requirements, for they are considered conservative, esthetic, and functional prosthetic options (Cardoso & Decurcio, 2018). Veneers provide adequate esthetics, registering a satisfactory clinical survival rate, color stability, vibrant appearance, and high resistance to abrasion and discoloration. A considerable number of studies show a survival rate of up to 20 years and a success level of 75%–100%. Dental ceramics consist of a vitreous phase (75%–80%), containing crystals inside; the presence of the true crystalline state of dental *ceramic* is limited to alumina Al_2_O_3_, the hardest and strongest known oxides (Sakaguchi & Powers, 2012).

One of the most widely used types of dental ceramics in manufacturing veneers is glass ceramic (Fu et al., 2020; Zanotto, 2010). Ivoclar Vivadent introduced this ceramic system in two forms: *IPS e.max CAD*, a block milled in CAD/CAM, and another using press‐lost wax, *IPS e.max Press*. The first generation of lithium disilicate, named *IPS Empress 2*, contained glass ceramic and was filled with glass particles, containing approximately 70% lithium disilicate. This was later presented as *IPS e.max*, where lithium silicate crystals play the role of filler crystals in this system as needle‐shaped crystals: 3–6 µm in length and 0.5–0.8 µm in diameter (Özdemir & Özdoğan, 2018). These crystals form regularly within the ceramic mold during the melting process; besides, glass‐reinforced lithium silicate is a ceramic that can be etched using hydrofluoric acid (Gresnigt et al., 2017).

One study indicates that lithium disilicate glass‐reinforced porcelain is less abrasive to opposite natural teeth, but more susceptible to wear than feldspar porcelain and glass reinforced with fluorapatite crystals. It displays higher elasticity compared to glass reinforced with fluorapatite crystals followed by feldspar porcelain and glass‐reinforced porcelain with Lucite crystals (Zhang et al., 2017). However, glass reinforced with lithium disilicate is considered highly translucent porcelain regardless of the high volume of crystals within its construction. This is due to the low refractive index of lithium disilicate crystals, reaching 1.55 (McLaren & Cao, 2009), which makes it indicated in cases of veneers and cosmetic anterior prostheses. It is also recommended for making anterior and posterior crowns and intracoronal fillings, as well as bridges with three dental units, provided that the lateral abutment does not exceed the second premolar (McLaren & Cao, 2009; Miwa et al., 2016).

As a result of the new development in zirconia and its structure and manufacturing methods, obtaining more translucent zirconia has proven feasible compared to the old generations, with improved phases of cosmetics. Such development made possible the use of zirconia in cosmetic areas with the help of external dyes and colorings to achieve color similarity to the adjacent natural teeth. This type of ceramic provides very acceptable esthetic outcomes in manufacturing veneers; experimentally, it reduces the risk of veneer fracture during clinical functioning (Borba et al., 2011; Calamia, 1996). Likewise, it is less costly compared to other materials. Zirconia structures provide a sufficient amount of shade to hide the discolored supports under them due to their high density (porosity < 0.05) and homogeneity even in limited thicknesses (Souza et al., 2018).

Our current investigation, henceforth, aims at comparing glass reinforced with crystals of lithium disilicate, *e.max*, to high translucent cer. This is held for manufacturing ceramic veneers, under the grounds of esthetic, functional, and biological aspects.

## 2 MATERIALS AND METHODS

### 2.1 Study design

This investigation follows the clinically controlled trials scheme. It was conducted at the Department of Operative Dentistry, the Faculty of Dental Medicine, at Damascus University. Furthermore, our research sample was distributed between the two groups after operationalizing the concept of randomization and blinding the researchers. The study included 12 patients who were divided into two groups:

- Group 1: 6 patients with 36 ceramic veneers manufactured from *IPS e.max* and applied on 6 anterior teeth.
- Group 2: 6 patients with 36 ceramic veneers manufactured from *high‐translucent zirconia* and applied on 6 anterior teeth.

Veneers were applied to 12 patients who complained of cosmetic problems, the age of whom ranged from 25–37 years. As well, each patient was randomly assigned to one of the two groups, *e.max* or *cubic zirconia*. Distribution blinding was done by using sealed envelopes, containing the number to refer to the assigned group. In addition to blinded distribution, two calibrating blinded observers made the assessment during the follow‐up periods: in case of any variation of results between them, a third assessment was sought to reach an accurate judgment.

*Inclusion criteria*:

a. age between 20 and 40 years
b. absence of abnormal functional habits such as bruxism
c. good condition of gingival and periodontal tissues
d. good oral health
e. a clear indication for patients for treatment with veneers: presence of diastema, poor dental restorations, slight modification of color in the discolored teeth, and modification of shape and sizes of the teeth
f. patient's written consent to enroll in the study and adherence to a follow‐up session

*Exclusion criteria*:

a. age under 18 years
b. bad oral hygiene
c. dysfunctional habits and bruxism
d. periodontal diseases
e. high risk of caries
f. inadequate enamel for bonding
g. extreme stinging and color change in tooth

*Research materials*:

1. Examination tools: periodontal probe with explorer, mouth mirror, and tweezer (Medesy)
2. Preparation: depth Marker diamond burs, diamond Taper round‐ended burs, finishing diamond burs (Komet) and Rubber Point kit, gingival retraction cord size (00–000) from (Ultradent), and impression materials (condensational silicon and additional silicon) (Zhermack)
3. Temporary crown and bridge material (ExperTemp; Ultradent)
4. Phosphoric Acid 37% and hydrofluoric acid 9% (Bisco)
5. MonoBond‐S (silane coupling agent) and light‐cured resin Cement—Milky Opaque, along with methacryloyloxydecyl dihydrogen‐phosphate (MDP) (Bisco).
6. Bonding agent (Ivoclar Vivadent)
7. Veneers manufacturing tools: CAD/CAM scanner from Zircodenta, tempering device, and Ivoclar Vivadent lithium disilicate glass‐reinforced ceramic heat injection furnace

### 2.2 Research procedure

To confirm that the patient meets the necessary criteria, a tissue examination was conducted. In addition, digital photographs and initial impressions were taken for the patient to review each clinical case and the cosmetic expectations for the treatment.

### 2.3 Preparation

Once the case was enrolled based on the inclusion criteria mentioned above, the treatment teeth were prepared to receive ceramic veneers. Loupes magnifiers were used under chair lighting to ensure a suitable probing depth for preparation and to standardize preparation criteria as much as possible. Minimal preparation was resorted to by adopting the butt joint pattern that guarantees shortening the cutting edge to obtain the required translucent and esthetic aspect. The incisal edge was prepared by depth guide bur; this was followed by preparing the cervical region 0.1–0.3 mm and the mid‐region 0.3–0.5 mm. The cutting edge was also reduced by 1.0–1.5 mm. After removing the enamel via a cone round‐end bur to reach the required depth, the finishing lines were put under the gingival area to guarantee the esthetic aspect and the emanating appearance, adopting the light chamfer scheme. Moreover, the preparation was extended to a completely labial surface without exceeding the interproximal contact, rounding all the edges and linear angles. Later, manufacturing the veneers was achieved following the exact laboratory steps for both the zirconia group and the e.max group (Baldissara et al., 2018; Daniele et al., 2022).

### 2.4 Impression

Following that stage, the impressions were made. First, a second gingival retraction was applied in the sulcular gingiva to provide an accurate thickness of the impression material. Appropriate impression trays for each patient were meticulously chosen, and additional silicon was used to make the impressions using the putty wash technique while taking an impression of the opposite side with alginate and a wax bite of the prepared teeth in a centric occlusion position. The selection of color was performed by a shade guide, and then the provisional restoration was made with the help of a silicone guide that was prepared before starting the whole process. The self‐cured intraoral acryl was injected into the silicone guide, then the provisional restoration was trimmed: the margins were well finished using the finishing burs. The impression was sent to the laboratory with information about the color and age of the patient and notes if that was necessary.

### 2.5 Bonding

This stage was divided into three phases.

1. Tooth‐related phase was commonly shared for the two groups. It included removing the provisional restoration and cleaning the surfaces of the teeth, after isolating the area by a rubber dam. The veneer parts were tried in the patient's mouth to ensure an appropriate fit. The final step was cleaning and etching the teeth surfaces by using 37% phosphoric acid, drying them thoroughly, and applying the bonding agent.
2. E.max‐related phase comprised treating the e.max's internal surface by etching it with 9% hydrofluoric acid, after which a silane layer was applied and yet another one to achieve shiny surfaces.
3. High‐translucent zirconia‐related phase encompassed sandblasting with particles of aluminum oxide coated with silica of the internal surfaces for the zirconia for 20 s (2.8 bar, 10 mm standoff distance). After drying, the application of a double bonding agent containing MDP for both types of restoration was done.

Finally, we put light‐cured resin cement for both types. A light cure was selected because it possesses more color stability than dual cure cement. The suggestion of utilizing light curing was introduced by Wassell et al. (2002); we applied it for only 4–5 s, but we eliminated the excessive cement with the help of a probe, using dental floss in approximal areas. Finally, the veneers were light‐cured from multiple angles to ensure complete polymerization. Following cementation procedures, the patients were instructed on oral hygiene measures.

### 2.6 Research indices

The following indices were used to evaluate the veneers according to predefined intervals of 1 week, 3 months, 1 year, and 3 years. The assessment was carried out according to Hickel. Indices contained surface and margin staining, color matching and translucency, presence of fracture or crack, contact points, patient's satisfaction, tooth vitality and hypersensitivity, and finally gingival response.

### 2.7 Clinical evaluation

Patients were assessed during the follow‐up sessions, and photographs were taken to document the condition at each follow‐up session. As for patients' satisfaction, a questionnaire was used to be filled out by the patients in each follow‐up session.

Surface and margin staining involved the evaluated surface and margin staining and color matching that were verified by the visual method comparing the shade taken immediately after the adhesive veneers and the shade during the follow‐up sessions. Fracture or crack was verified by a visual exam by mirror and illumination at the chairside. The contact points were investigated by the difficulty of passing a coated dental floss through the interdental contact points. Additionally, patients' satisfaction was measured by asking patients to fill out a questionnaire as mentioned previously. Tooth vitality was monitored by examining the vitality of abutment and pulp sensitivity by a pulp‐vitality spray tester and comparing it with adjacent teeth. Lastly, the gingival response was traced by measuring the periodontal sulcus in the follow‐up session and comparing it to the initial state.

### 2.8 Statistical analysis

The collected data were analyzed using SPSS at the level of significance that was set to *p* < 0.05. Other statistical tests, such as Mann–Whitney *U* and Kruskal–Wallis, were used to compare the research groups and variables.

## 3 RESULTS

**Figure 1:**
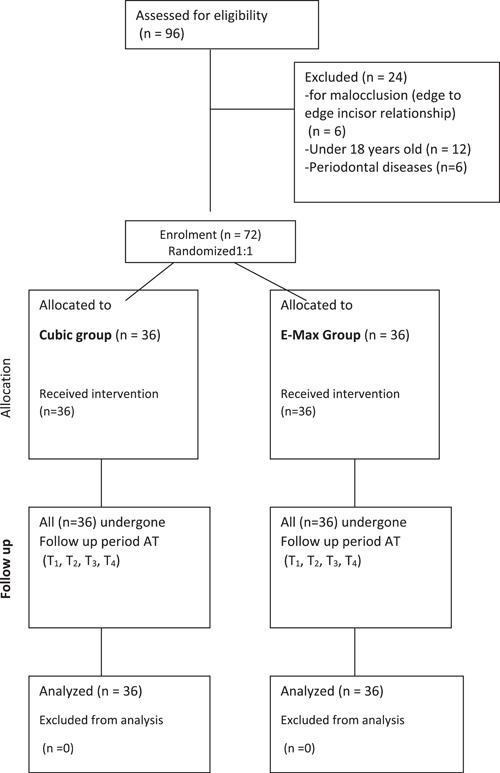
Consort diagram of the study sample.

A total of 16 participants were examined, but the final sample size consisted of 12 patients and included 72 samples as seen in Figure 1. The sample included 2 males (16.6%) and 10 females (83.3%) whose ages ranged between 25 and 37 years, with a mean of age of 28.8 and a standard deviation of 4.2 years.

Table 1 shows descriptive and inferential statistics for the research indices to determine if there is a significant difference between the two groups, zirconia, and e.max, after 1 week, 3 months, 1 year, and 3 years. The results showed that there was no staining on the surface for the two groups, except for 2 cases from the e.max group (6.25%) after 3 months, 12 cases (37.5%) after 3 years, and 6 cases (18.75%) from the zirconia group. There exists no significant difference between the two groups. In addition, there is no marginal staining for the two groups.

**Table 1:** Descriptive and inferential statistical analysis for the research indices during measurement times.

				Count	Percentage	*p* Value	Significance
Surface staining index	Zirconia	Surface staining after a week	No surface staining	32	100.0%	1.000 After a week, 1 year 0.29 After 3 months 0.1 After 3 years	No significant difference
		Surface staining after 3 months	No surface staining	32	100.0%		
		Surface staining after 1 year	No surface staining	32	100.0%		
		Surface staining after 3 years	No surface staining	26	81.25%		
			Simple surface staining	6	18.75%		
	E.max	Surface staining after a week	No surface staining	32	100.0%		
		Surface staining after 3 months	No surface staining	30	93.75%		
			Simple surface staining	2	6.25%		
		Surface staining after 1 year	No surface staining	32	100.0%		
		Surface staining after 3 years	No surface staining	20	62.5%		
			Simple surface staining	12	37.5%		
Edges staining	Zirconia	Edges staining after a week	No edges staining	32	100.0%	1.0000 All timeframe	No significant difference
		Edges staining after 3 months	No edges staining	32	100.0%		
		Edges staining after 1 year	No edges staining	32	100.0%		
		Edges staining after 3 years	No edges staining	32	100.0%		
	E.max	Edges staining after a week	No edges staining	32	100.0%		
		Edges staining after 3 months	No edges staining	32	100.0%		
		Edges staining after 1 year	No edges staining	32	100.0%		
		Edges staining after 3 years	No edges staining	32	100.0%		
Color matching	Zirconia	Color matching after a week	Color matching is good	32	100.0%	1.000 All timeframe	No significant difference
		Color matching after 3 months	Color matching is good	32	100.0%		
		Color matching after 1 year	Color matching is good	32	100.0%		
		Color matching after 3 years	Color matching is good	32	100.0%		
	E.max	Color matching after a week	Color matching is good	32	100.0%		
		Color matching after 3 months	Color matching is good	32	100.0%		
		Color matching after 1 year	Color matching is good	32	100.0%		
		Color matching after 3 years	Color matching is good	32	100.0%		
Crack or fracture	Zirconia	Crack or fracture after a week	No crack or fracture	32	100.0%	1.000 All timeframe	No significant difference
		Crack or fracture after 3 months	No crack or fracture	32	100.0%		
		Crack or fracture after 1 year	No crack or fracture	32	100.0%		
		Crack or fracture after 3 years	No crack or fracture	32	100.0%		
	E.max	Crack or fracture after a week	No crack or fracture	32	100.0%		
		Crack or fracture after 3 months	No crack or fracture	32	100.0%		
		Crack or fracture after 1 year	No crack or fracture	32	100.0%		
		Crack or fracture after 1 year	No crack or fracture	32	100.0%		
		Crack or fracture after 3 years	No crack or fracture	32	100.0%		
Contact points	Zirconia	Contact points after a week	Contact points are normal	32	100.0%	1.000 All timeframe	No significant difference
		Contact points after 3 months	Contact points are normal	32	100.0%		
		Contact points after 1 year	Contact points are normal	32	100.0%		
		Contact points after 3 years	Contact points are normal	32	100.0%		
	E.max	Contact points after a week	Contact points are normal	32	100.0%		
		Contact points after 3 months	Contact points are normal	32	100.0%		
		Contact points after 1 year	Contact points are normal	32	100.0%		
		Contact points after 3 years	Contact points are normal	32	100.0%		
Patient satisfaction	Zirconia	Patient satisfaction after a week	Patient is completely satisfied	32	100.0%	1.000 All timeframe	No significant difference
		Patient satisfaction after 3 months	Patient is completely satisfied	32	100.0%		
		Patient satisfaction after a year	Patient is completely satisfied	32	100.0%		
		Patient satisfaction after 3 years	Patient is completely satisfied	32	100.0%		
	E.max	Patient satisfaction after a week	Patient is completely satisfied	32	100.0%		
		Patient satisfaction after 3 months	Patient is completely satisfied	32	100.0%		
		Patient satisfaction after a year	Patient is completely satisfied	32	100.0%		
		Patient satisfaction after 3 years	Patient is completely satisfied	32	100.0%		
Vitality and hypersensitivity	Zirconia	Tooth vitality after a week	Normal vitality	20	62.5%	0.61 After a week 1.000 After 3 months, after 1 year, after 3 years	No significant difference
			Simple hypersensitivity	12	37.5%		
		Tooth vitality after 3 months	Normal vitality	32	100.0%		
		Tooth vitality after 1 year	Normal vitality	32	100.0%		
		Tooth vitality after 3 years	Normal vitality	32	100.0%		
	E.max	Tooth vitality after a week	Normal vitality	18	56.25%		
			Simple hypersensitivity	14	43.75%		
		Tooth vitality after 3 months	Normal vitality	32	100.0%		
		Tooth vitality after 1 year	Normal vitality	32	100.0%		
		Tooth vitality after 3 years	Normal vitality	32	100.0%		
Gingival inflammation	Zirconia	Gingival inflammation after a week		32	.00	1.000 All timeframe	No significant difference
		Gingival inflammation after 3 months		32	.00		
		Gingival inflammation after a year		32	.00		
		Gingival inflammation after 3 years		32	.00		
	E.max	Gingival inflammation after a week		32	.00		
		Gingival inflammation after 3 months		32	.00		
		Gingival inflammation after a year		32	.00		
		Gingival inflammation after 3 years		32	.00		

With respect to de‐bonding occurrences, the result of our study showed that there were no de‐bonding events in both groups at all subsequent sessions.

A good color match was observed for both of the groups during all measurement intervals, and there was no significant difference between the two groups. There was no fracture or crack in the edges during the measurement times for the two groups, and there was no significant difference between them. Furthermore, it was detected that there were normal contact points during the measurement times for both groups, and there was no significant difference between them. Patients were completely satisfied with the prostheses in both research groups, and there was no statistically significant difference between the two groups.

With regard to tooth vitality, a minor hypersensitivity was observed in 37.5% of the cases in the zirconia group after a week, in comparison to 43.75% of the cases in the e.max group after a week. However, the vitality was normal for both groups after 3 months, with no significant difference between the two groups during the measurement times. Finally, no periodontal inflammation was detected across the research participants during the measurement times.

## 4 DISCUSSION

Ceramic with high crystal content, such as yttria‐stabilized tetragonal zirconia polycrystal (TZP‐Y), is primarily engaged in manufacturing structures of fixed crowns and bridges because of its high fracture toughness properties. Recently, conversely, zirconia has witnessed a plethora of changes to its microstructure, which increased its translucency without losing its fracture resistance. Henceforth, its clinical utilizations are expanded. Consequently, translucent zirconia is widely considered to be an esthetic material, particularly in making anterior and posterior crowns and bridges, along with veneers.

This type of ceramic has been found to offer incredibly satisfactory esthetic outcomes when used in veneers; it reduces the risk of layered porcelain fracture throughout clinical applications and experimentation. TZP‐Y affords lower cost compared to other used materials; zirconia structures provide sufficient areas of shading to hide the underlying discolored abutment due to its high density and homogeneity even when the region thicknesses are scarce. Hence, the idea of research revolved originally around evaluating the clinical performance of veneers manufactured from both e.max and high‐translucency zirconia. The investigators of this current study faced limited clinical research in the medical literature addressing the use of zirconia in veneer manufacture. Such a shortcoming was accompanied by another lack of clinical research that studied the clinical evaluation of the esthetic, functional, and biological aspects of veneers also manufactured from these two materials, namely the *e.max* and the *highly translucent zirconia*. The clinical performance of veneers was evaluated according to the International Dental Federation (FDI) criteria. This is due to the fact that these criteria are comprehensive and cover all the esthetic, functional, and biological aspects. Our present study included three esthetic criteria (surface discoloration, margins discoloration, and color matching), three functional criteria (veneers' fracture or crack, anatomical shape of contact points, and patient's satisfaction), and biological criteria (tooth vitality, posttreatment hypersensitivity, and gingival response). Each of these criteria has been expressed on a scale of 5 degrees.

Ceramic veneer failure can be categorized as either absolute or relative. Absolute failure is when there are irreparable cracks that make the veneer unacceptable and require a complete remake. Relative failure, on the other hand, is when there are cracks that are still clinically acceptable or when the ceramic attachment fails but can be rebonded (Guess & Stappert, 2008).

One common issue in dentistry is bonding failure when using resin cement to attach ceramic veneers, regardless of whether the veneers are made of e‐max ceramic or cubic zirconia. This can be caused by various factors, such as microleakage, secondary carriers resulting from the dissolution of resin cement, and the dissolution of bonding agents due to chemical and mechanical factors within the oral cavity (Huang et al., 2018). Additionally, bonding failure can occur when there are errors in handling the dental or prosthesis surface (Morita et al., 2016).

On the other hand, the effectiveness and polymerization time of a light cure device has a significant impact on the curing process of resin cement (Rasetto et al., 2004). Additionally, the fillers present in the resin cement play a crucial role in determining the efficacy of the light‐curing device. Fine fillers, in particular, are more adept at scattering light as compared to hybrid fillers (Rueggeberg et al., 1994). Finally, de‐bonding also occurs when a ceramic piece breaks off due to high occlusion forces and nonfunctional habits (Radz, 2011).

In our research, we noted that there were no de‐bonding occurrences in either group. This could be attributed to following an accurate cementation protocol, and case selection which had a good occlusion and a precise fit of the ceramic veneers onto the enamel surfaces, which exhibited a comparable elastic modulus to the veneers. Moreover, the exceptional robustness and mechanical precision of the glass ceramics, fortified with lithium disilicate crystals and zirconia porcelain, further amplified these outcomes.

When evaluating the surface discoloration criteria in this study, no discoloration was observed in the veneers' surface in both study groups (e.max and zirconia), except for two veneers in the (e.max) group after 3 months of observation. The discoloration was simple and could easily be removed by polishing: the surface of e.max veneers was more discolored compared with the zirconia surface.

No discoloration was observed in the veneers' margins in both study groups through the observation periods. This agrees with Ozel and Aykor's study, in which it was found that there was no marginal discoloration of lithium disilicate crystal (e.max) veneers (Nejatidanesh et al., 2018). In addition, it agrees with Ahn et al. (2019) who observed six anterior veneers for 6 months, and no deboning or staining was observed (Ahn et al., 2019). Contrastively, this study disagreed with Aslan's long‐term study, which traced 11.7% of marginal discoloration in lithium disilicate glass‐ceramic veneers (Aslan et al., 2019). The difference in these percentages is perhaps due to the difference in the observation period between these studies; it might also be ascribed to the marginal fit quality, use of light‐cured resin cement in the adhesion, marginal finishing quality, and patient adequate oral hygiene. Nonetheless, only one case of marginal discoloration was observed in another study (Peláez et al., 2012), which employed translucent zirconia bridges to replace the second premolar or the first molar.

Color matching and translucency were good in both of the study groups, namely e.max and zirconia. The results of our study disagreed with Nejatidanesh et al., who found that a large percentage of the veneers (IPS e.max) had discoloration over time. The reason was attributed to the fact that the discoloration occurred on the adjacent natural teeth, not on the veneers (Nejatidanesh et al., 2018). Another study comparing *IPS e.max Press* to *monolithic zirconia*, in the form of discs, concluded that lithium disilicate claims more color stability over monolithic zirconia (Kurt & Turhan Bal, 2019).

The bonding to zirconia has been a focus of scientific research during the past period. Various treatment methods have been used: sandblasting using aluminum oxide, nanostructured alumina coating, tribochemical silica coating followed by silanization, and application of resin cement containing 10‐MDP monomer, among others. In this current study, there were no cases in which fracture or loss of veneer bonding was verified in either of the study groups. The results of this study come in accordance with Nejatidanesh et al. They did not record any fracture or cracking in the e.max veneers; this was ascribed to the high flexural strength of the material used, which can reach up to 420 MPa (Nejatidanesh et al., 2018) and does not have any problem in adhesion and no accident trauma was recorded. In addition, this finding agrees with other studies that reported a high success rate when they used dense zirconia (Klink & Hüttig, 2016; Sailer & Hämmerle, 2014). Also, this agrees with Matthias Kern's study, which found there were no fracture events in cases of zirconia ceramic; this is due to the fact that zirconia has twice the flexural strength than glass infiltrated alumina (Kern et al., 2017). What's more, the contact points were normal in all veneers in the two groups during each observation interval. This is perhaps linked to two factors: the expertise of the dental technician and the accuracy of the sculpting devices in forming natural, esthetic, and anatomical proximal areas. Nevertheless, the patients expressed their satisfaction with the shape, color, and verbal function in both study groups (e.max and zirconia) during the observation periods. Diagnostic waxing and obtaining the patient's consent before starting the process might have contributed to this outstanding acceptance. In addition to that, the prompt manufacture of prostheses, with the help of the diagnostic waxing, allowed the patient to test pronunciation, as well as the shape before the final prosthesis was made. Hence, our study agrees with Aslan et al. (2019), which achieved esthetic satisfaction with 98% for glass ceramic reinforced with lithium disilicate crystals (e.max) veneers. It also agrees with Liu et al. who conducted their investigation on glass ceramic reinforced with lithium disilicate crystals (e.max) veneers. Their patients' satisfaction with esthetic aspects was evaluated using the VAS scale; it showed the mean of patients' satisfaction with the esthetic aspect to be 9.2 during the 2‐year span of observation (Liu et al., 2012).

Our results also agreed with Nejatidanesh et al. (2018), indicating that dental sensitivity following the cementation of veneers gradually disappeared over time, whereas this study differed from other studies that did not record any sensitivity after adhering veneers (Sun et al., 2013; Yuce et al., 2019). This finding may be attributed to the fact that cementation and preparation produce subjective sensations of pain related to the patient. However, no gingival inflammation was observed in the study groups during all the observation intervals. The results of our study agreed with Nejatidanesh et al. (2018), for they did not observe an increase in the gingival index over time. It also agreed with D'Arcangelo et al. (2012), which did not show an increase in the gingival index when using veneers. The reason is mainly ascribed to the persistence of preparation in the cervical area, quality of adhesion and marginal finishing, good marginal fitting of the prostheses, as well as the patients' commitment to oral care and follow‐up sessions.

## 5 CONCLUSION

Within the limitation of an extensive follow‐up period, we can conclude the following:

1. Zero de‐bonding event occurred at 3 years follow‐up in the cubic zirconia group and e.max group.
2. There was a simple hypersensitivity after a week of adhesion in both comparison groups, but it disappeared in follow‐up sessions.
3. There is a simple surface staining after 3 years follow‐up in the test and control groups.

## AUTHOR CONTRIBUTIONS

Hiba A. Fawakhiri developed the idea, designed the research, wrote the manuscript, analyzed the data, and finalized the manuscript. Shaza Kanout and Souad Abboud supervised the research and helped in writing the manuscript.

## CONFLICT OF INTEREST STATEMENT

The authors declare no conflict of interest.

## ETHICS STATEMENT

Approval and consent to participate were meticulously met. Ethical approvals were obtained from both the Higher Committee for Medical Research at Damascus University and from the Scientific Research Committee at the Faculty of Dental Medicine, Damascus University (N: 1059 t/m date: 15‐12‐2018). Moreover, all methods were carried out in accordance with relevant guidelines and regulations. Informed consent was obtained from all participants before participating in the research.

## Data Availability

Data underlying this manuscript will be shared on reasonable request to the corresponding author.
